# Gut microbiota carbon and sulfur metabolisms support *Salmonella* infections

**DOI:** 10.1093/ismejo/wrae187

**Published:** 2024-10-15

**Authors:** Ikaia Leleiwi, Katherine Kokkinias, Yongseok Kim, Maryam Baniasad, Michael Shaffer, Anice Sabag-Daigle, Rebecca A Daly, Rory M Flynn, Vicki H Wysocki, Brian M M Ahmer, Mikayla A Borton, Kelly C Wrighton

**Affiliations:** Department of Cell and Molecular Biology, Colorado State University, Plant Sciences Bldg. 307 University Ave, Fort Collins, CO 80523, United States; Department of Soil and Crop Sciences, Colorado State University, Plant Sciences Bldg. 307 University Ave, Fort Collins, CO 80523, United States; Department of Soil and Crop Sciences, Colorado State University, Plant Sciences Bldg. 307 University Ave, Fort Collins, CO 80523, United States; Department of Microbiology, Immunology, and Pathology, Microbiology Building, 1682 Campus Delivery Colorado State University, Fort Collins, CO 80523, United States; Department of Chemistry and Biochemistry, The Ohio State University, 200 CBEC Building 151 W. Woodruff Ave. Columbus, OH 43210, United States; Department of Chemistry and Biochemistry, The Ohio State University, 200 CBEC Building 151 W. Woodruff Ave. Columbus, OH 43210, United States; Department of Soil and Crop Sciences, Colorado State University, Plant Sciences Bldg. 307 University Ave, Fort Collins, CO 80523, United States; Department of Microbial Infection and immunity, The Ohio State University, 776 Biomedical Research Tower, 460 W. 12th Avenue, Columbus, OH 43210-2210, United States; Department of Soil and Crop Sciences, Colorado State University, Plant Sciences Bldg. 307 University Ave, Fort Collins, CO 80523, United States; Department of Soil and Crop Sciences, Colorado State University, Plant Sciences Bldg. 307 University Ave, Fort Collins, CO 80523, United States; Department of Chemistry and Biochemistry, The Ohio State University, 200 CBEC Building 151 W. Woodruff Ave. Columbus, OH 43210, United States; Resource for Native Mass Spectrometry Guided Structural Biology, The Ohio State University, 280 Biomedical Research Tower 460 W. 12th Ave. Columbus, OH 43210, United States; Department of Microbial Infection and immunity, The Ohio State University, 776 Biomedical Research Tower, 460 W. 12th Avenue, Columbus, OH 43210-2210, United States; Department of Soil and Crop Sciences, Colorado State University, Plant Sciences Bldg. 307 University Ave, Fort Collins, CO 80523, United States; Department of Cell and Molecular Biology, Colorado State University, Plant Sciences Bldg. 307 University Ave, Fort Collins, CO 80523, United States; Department of Soil and Crop Sciences, Colorado State University, Plant Sciences Bldg. 307 University Ave, Fort Collins, CO 80523, United States; Department of Microbiology, Immunology, and Pathology, Microbiology Building, 1682 Campus Delivery Colorado State University, Fort Collins, CO 80523, United States

**Keywords:** Microbiome, murine, lactate, lactic-acid bacteria, metatranscriptomics, genome-resolved

## Abstract

Salmonella enterica serovar Typhimurium is a pervasive enteric pathogen and ongoing global threat to public health. Ecological studies in the *Salmonella* impacted gut remain underrepresented in the literature, discounting microbiome mediated interactions that may inform *Salmonella* physiology during colonization and infection. To understand the microbial ecology of *Salmonella* remodeling of the gut microbiome, we performed multi-omics on fecal microbial communities from untreated and *Salmonella*-infected mice. Reconstructed genomes recruited metatranscriptomic and metabolomic data providing a strain-resolved view of the expressed metabolisms of the microbiome during *Salmonella* infection. These data informed possible *Salmonella* interactions with members of the gut microbiome that were previously uncharacterized. *Salmonella-*induced inflammation significantly reduced the diversity of genomes that recruited transcripts in the gut microbiome, yet increased transcript mapping was observed for seven members, among which *Luxibacter* and *Ligilactobacillus* transcript read recruitment was most prevalent. Metatranscriptomic insights from *Salmonella* and other persistent taxa in the inflamed microbiome further expounded the necessity for oxidative tolerance mechanisms to endure the host inflammatory responses to infection. In the inflamed gut lactate was a key metabolite, with microbiota production and consumption reported amongst members with detected transcript recruitment. We also showed that organic sulfur sources could be converted by gut microbiota to yield inorganic sulfur pools that become oxidized in the inflamed gut, resulting in thiosulfate and tetrathionate that support *Salmonella* respiration. This research advances physiological microbiome insights beyond prior amplicon-based approaches, with the transcriptionally active organismal and metabolic pathways outlined here offering intriguing intervention targets in the *Salmonella-*infected intestine.

## Introduction

Non-typhoidal *Salmonella*, including *Salmonella enterica* serovar Typhimurium (hereafter *Salmonella*), cause more than 1.3 million infections and hundreds of deaths annually in the United States [1, 2]. The global burden of non-typhoidal *Salmonella* is magnitudes larger and the World Health Organization reports 550 million infections per annum [3]. Given the global emergence of multidrug-resistant *Salmonella* strains [4–6], there is an urgency to develop alternatives to traditional antibiotic therapy, which could leverage understanding of the metabolic targets that sustain pathogen infection.

Much of the prior research on *Salmonella* metabolism during infection was done using gnotobiotic mice, or in mice models that require pre-treatment with antibiotics for *Salmonella* establishment [7–11]. Such models negate natural microbiota interactions during infection. These prior studies often individually demonstrated that *Salmonella* can harness host inflammation and a range of respiratory electron acceptors present in the gut during infection, including oxygen, oxidized nitrogen, and oxidized sulfur species [9, 12, 13]. It was also shown that *Salmonella* utilizes microbiota-derived carbon sources like succinate, 1,2 propanediol, and ethanolamine [8, 14–16]. Additionally, prior studies demonstrated that *Salmonella* utilized host-derived lactate and tetrathionate as a carbon source and electron acceptor respectively [7, 9]. Although these foundational findings into *Salmonella* metabolism, highlighted in Supplementary table 1, laid an important foundation of physiological processes germane to *Salmonella* expansion and persistence in the gut [17–23], there is limited work published on the post-colonization microbiota metabolic interactions with *Salmonella*. Further, these metabolisms have not been collectively examined through community-wide transcriptome-based analyses, and until now there has been no holistic evaluation of *Salmonella* sulfur and carbon metabolism amidst an unperturbed microbiome.

The CBA mouse offers a model for observing *Salmonella* gastroenteritis effects on the gut microbiome, as it avoids pre-treatment with antibiotics and occludes *Salmonella* systemic infection [24, 25]. Importantly, this disease model mimics the inflammatory response to *Salmonella* in the human gastrointestinal tract and is becoming a mainstay of *Salmonella* research [8, 12, 18, 26–28]. In our prior work with CBA mice we repeatedly showed that *Salmonella* significantly restructured the microbial community of the gut, reducing the relative abundance of many dominant *Clostridia* and *Bacteroidia*, while enriching for rare *Bacilli* and selected members of the *Clostridia, Bacteroidia*, and *Verrucomicrobiae* [29, 30]. Previously, we created a metagenome assembled genome (MAG) catalog for the CBA mouse model (CBAJ-DB), sampling over 2000 MAGs from *Salmonella* infected and non-infected mice [29]. The CBAJ-DB revealed unique bacterial lineages from *Bacilli, Bacteroidia,* and *Clostridia* not found in microbiome catalogs from other mouse strains. We showed *Salmonella*-impacted microbial communities also had a greater potential for respiration and lower potential for butyrate production [29].

Though important in recovering genomic representatives of microbial lineages that could withstand and were often enriched during *Salmonella* infection, our prior work was entirely DNA based inference. This limited our ability to expound pertinent *Salmonella* metabolic processes and obscured possible metabolic exchanges between *Salmonella* and other gut members. Here we utilized the CBAJ-DB to contextualize paired metabolome and metatranscriptome data from non-infected and *Salmonella* infected CBA/J mice. First, we profiled *Salmonella* gene expression during the late stage of infection, providing insights into overrepresented metabolic transcripts during *Salmonella* infection. Our multi-omics approach interrogated metabolic pathways transcribed during infection, revealing new microbial contributions to carbon and sulfur cycling. This included possible re-assignment of metabolites historically considered only host derived, such as lactate from host epithelia starved of butyrate [7]. Ultimately, multi-omics allowed renewed examination of the ecology during pathogen infection in a robust community context, providing a detailed look at the microbiome interaction framework.

## Materials and methods

### Strains and media

*S. enterica* serovar Typhimurium strain 14 028 (*S. typhimurium* 14 028) cultures were washed and resuspended in water after overnight incubation in Luria-Bertani broth at 37°C with constant agitation.

### Animals and experimental design

Female CBA (Bagg albino female × Dilute Brown Agouti male) mice were procured from The Jackson Laboratory (Bar Harbor, ME). Female mice were used exclusively in this study as they could be co-housed within cages. Mice were randomly selected to populate cages and were kept five per cage in conventional enclosures in a temperature controlled 12-h light/dark cycle. Irradiated mouse chow (Teklad, 7912) was made available *ad libitum* to all mice. Cages were assigned treatment groups, with mice in the infected cages inoculated with 10^9^ CFU S. typhimurium 14 028 via oral gavage on day 0 with no subsequent treatment. Uninfected mice were left with no treatment. Animal experiment protocol was approved by The Ohio State University Institutional Animal Care and Use Committee (IACUC; OSU 2009A0035).

**Figure 1:**
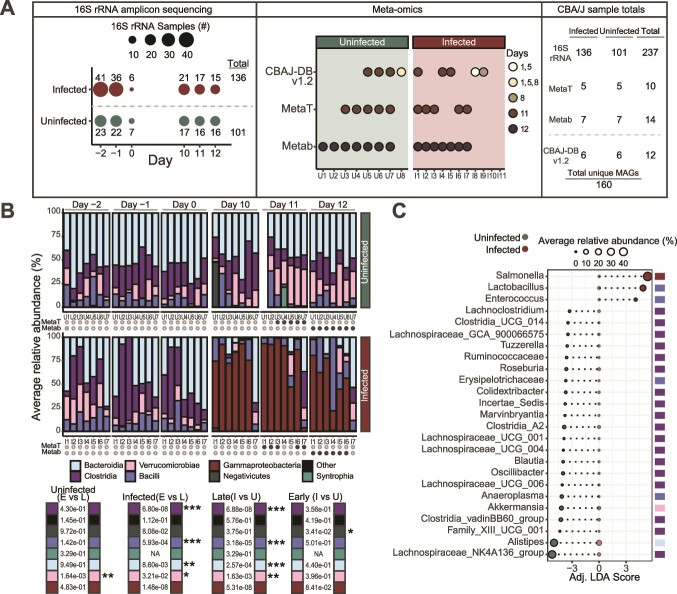
***Salmonella* infection enriches for distinct bacterial community membership.** (**A**) experimental sampling scheme showing total fecal samples taken on each day for 16S rRNA gene amplicon sequencing (left), and multi-omics analysis (middle). *Salmonella* treatment start is on day 0 and specific sample totals by treatment are listed in the right figure panel. Mice with multi-omics data are designated with a “U” if they were in the uninfected group (n = 8) or with an “I” if they were in the *Salmonella* infected group (n = 11). Samples used to reconstruct genomes for the CBAJ-DB v1.2 were collected from either day 1, 5, 8, or 11 as shown by the top row of circles in the middle panel. Unique MAGs (n = 160) listed in the right panel are all medium or high quality (contamination <10% and completeness ≥50%). (**B**) stacked bars showing the ASV class distribution of mice that have metatranscriptomic and metabolomic data. Mouse U1 day 11 sample was omitted via ASV table filtering (see methods). Significant differences (Wilcoxon rank sum) between classes of either early timepoints (E; days −2, −1, and 0), late time points (L; days 10, 11, and 12), infected samples (I), or uninfected samples (U). Colored circles below the bars represent either metatranscriptomics or metabolomics was performed on that particular mouse and the circle color denotes the treatment (green for uninfected and red for infected). (**C**) linear discriminant analysis of 16S rRNA gene amplicon data from late timepoint samples (days 10–12) from the subset of mice with metatranscriptomic and metabolomic data. Points are sized by relative abundance of each genus within a treatment (infected or uninfected) and are colored by treatment where points aligning with x-axis value 0 are the relative abundance of each genus in the non-significant treatment. Genera classes are listed by color on the right of the plot.

Mouse fecal pellets were collected from 38 mice before and after treatment initiation (on days −2, −1, 0, 10, 11, or 12) on autoclaved aluminum foil. Sample details by mouse are outlined (Supplementary Data File S1, Fig. 1). Fecal pellets were immediately placed in labeled microcentrifuge tubes and flash frozen with EtOH/dry ice prior to storage at −80°C until further processing. Fecal material was selected here as the sample type due to a prior study [30] where it was found the membership of the fecal and cecal communities during *Salmonella* infection could not be discerned statistically. Additionally, compared to the feces, cecal samples had poor microbiota transcript recovery because of high mouse background due to high levels of cecal inflammation [30].

### Lipocalin-2 measurement

Fecal samples were vortexed for 20 min in PBS containing 0.1% Tween 20 (100 mg/ml). Resulting suspensions were centrifuged at 12 000 rpm for 10 min at 4°C and the supernatant was used to measure levels of inflammation marker Lipocalin-2 using the Duoset murine Lcn-2 ELISA kit (R&D Systems, Minneapolis, MN). Difference in mean lipocalin-2 levels between groups was determined with Dunn’s test of multiple comparisons in R using dunn.test from the FSA (v0.9.5) package.

### DNA/RNA extraction and sequencing

All nucleic acid extraction for 16S rRNA gene amplicon sequencing was performed using ZymoBIOMICS DNA/RNA Miniprep Kit (Zymo Research) and stored at −20°C until further processed. PCR amplification of the V4 hypervariable region of 16S rRNA gene was performed using 30 cycles and unique sequence barcodes in each primer were used to identify multiplexed samples. Both primers (universal 515F and 806R) contained sequencer adapter regions. Total nucleic acid extraction and sequencing for metagenomics was performed as previously described [29] at the Genomics Shared Resource facility at Ohio State University. Metatranscriptomic RNA extraction and isolation was performed on feces from 10 mice sampled 11 days post infection with Zymo-Seq Ribo Free Total RNA Library Kit Cat No. R3000 and 2 × 151 bp paired end reads were produced from corresponding cDNA at UC Denver Sequencing Facility using a HiSeq 2500 Sequencing System (Illumina).

### Metabolite sample preparation and analysis

1 ml of a solution composed of three different solvents (water/methanol/dichloromethane, 1/2/3, v/v/v) was used for fecal metabolite extraction, followed by physical disruption with a sonicator (Bioruptor, Diagenode, Belgium). The disrupted fecal suspension was vortexed and incubated at room temperature. The top aqueous layer was transferred, dried under vacuum, and reconstituted for metabolomics analysis.

The Agilent 6545 QTOF mass spectrometer equipped with Agilent 1290 UHPLC systems liquid chromatography was used for an untargeted metabolomics study on mouse fecal pellet extractions (n = 13) from day 12. To improve the metabolite coverage, the prepared fecal samples were subjected to reversed-phase liquid chromatography with C18 column (ACQUITY UPLC HSS T3 1.8 μm, 2.1 × 100 mm, Waters Corporation, MA, USA) and hydrophilic interaction liquid chromatography (HILIC, ACQUITY UPLC BEH HILIC 1.7 μm 2.1 × 150 mm, Waters Corporation, MA, USA). Water with 0.1% formic acid (A) and acetonitrile with 0.1% formic acid (B) were used for reversed-phase separation. The flow rate was set at 0.3 ml /min with the gradient as follows: 2% B for 0–2 min, from 2% B to 30% B for 4 min, to 50% B for 8 min, and 98% B for 1.5 min and held at 98% B for 1 min, then returning into initial gradient for equilibrium for 1.5 min. For HILIC separation, Water/acetonitrile (95/5) with 0.1% formic acid and 10 mM ammonium formate (A) and water/acetonitrile (5/95) with 0.1% formic acid and 10 mM ammonium formate (B) were prepared. For gradient elution, 99% B was held for 2 min, gradually reduced to 75% B for 7 min and reduced again to 45% B for 5 min. And the gradient was held at 45% B for 2 min, and returned to 99% B. The flow rate was set at 0.3 ml /min. The quality control (QC) sample was prepared by mixing an equal volume of each sample. The QC sample was analyzed after every six samples.

The collected mass spectra were converted into mzML format with MSconvert in Proteowizard [31]. The data analysis for untargeted, global profiling experiments was performed with Progenesis QI (Waters Corporation/Nonlinear Dynamics). The Compound MS/MS library used in the study for metabolite annotation was the embedded Progenesis database with METLIN/Waters collaboration for fragmentation patterns, human metabolome database (HMDB), *Escherichia coli* metabolome database (ECMDB), and Lipid Maps. All annotated metabolites were manually inspected to be reported and metabolite significance between treatments was determined with ANOVA (Supplementary Data 4).

All metabolite samples were included in the ANOVA significance calculation (uninfected n = 7, infected n = 7). Subsequent analyses omitted the metabolome from mouse I7 in the infected group because all three technical replicates for this infected mouse were outside the range for both treatment groups, and thus it was deemed an outlier. Any missing values in the remaining metabolite data were imputed with half the smallest value recorded for each feature. Data were log transformed and Pareto scaled [32] and principal component analysis was performed in R version 4.1.3 with the prcomp function in the vegan package (v2.6–4). PERMANOVA was performed on Bray-Curtis distances calculated from untransformed data using the vegdist function from vegan. Euclidian distances from the centroid were calculated for important (threshold >0.09 & < −0.09) loadings in PC1 and PC2 (see script Metab_pca.R).

### 16S rRNA gene analysis

Amplicon sequencing fastq data generated for this experiment were a subset of data encompassing days −2, −1, 0, 10, 11, and 12 from a larger experiment under Bioproject number PRJNA348350 and were processed in a QIIME2 2019.10.0 environment, with reads demultiplexed and then denoised with DADA2 [33, 34]. For all sequencing runs (n = 6), forward reads were truncated at 246 bps and reverse reads were truncated at 167 bps. Feature tables from each sequencing run were combined and ASVs were later assigned taxonomy with the silva-138-99-515-806-nb-classifier in a QIIME2 2022.8.0 environment [33, 35]. The resulting ASV table was filtered with R version 4.1.3 to include only samples from days within the scope of this paper and to remove any samples with zero counts for every ASV (n = 2 samples). Next, ASVs were removed with zero counts in every remaining sample (n = 22,175 ASVs), and subsequently samples with fewer than 1000 counts across all remaining ASVs were dropped (n = 3 samples). Finally, any ASVs designated as mitochondria, chloroplast, unassigned at the domain level, or assigned Eukaryota were removed (n = 66 ASVs). The 16S rRNA V4 region from *S. enterica* Typhimurium ATCC 14028 reference genome was manually aligned with Geneious Prime 2020.1.2 to a single *Enterobacteriaceae* ASV (ASV ID = 4cbfff144d4e7a4e0f4619ed505be070) with 100% sequence identity to confirm taxonomy as *Salmonella*. The designation “high responder” was applied to any mouse with *Salmonella* ASV relative abundance ≥25% in at least one sample. Any mouse from the infected group was not included in the analysis unless it was a high responder. The resulting feature table contained 1405 unique ASVs (Supplementary Data File S1).

### 16S rRNA statistical analysis

*ASV Community Metrics and Class Significance.* ASV communities from mice with metabolomics data and metatranscriptomics data (n = 14) were categorized by sampling day: Early (day 2 – day 0) and Late (day 10 – day 12). Average ASV relative abundance within each class was calculated and the eight most abundant classes were retained and any other ASVs were classified as “Other”. Significant differences between classes and treatments were calculated with the Mann–Whitney U test in R.

*16S rRNA Linear Discriminant Analysis.* The filtered ASV table was limited to samples (n = 41) from mice that also had metabolomics or metatranscriptomic data including only samples from days 10, 11, and 12. ASVs with ambiguous genus designations were removed and then relative abundance of each ASV was calculated within samples. The table was collapsed to the genus level and linear discriminant analysis was performed with LEfSe to determine important taxa from each treatment [36].

*ASV High-Responder Correlation Network.* First the filtered ASV feature table was collapsed to the genus level and features were removed with 5000 or fewer counts and ambiguously named genera were removed (“uncultured”, “uncultured bacterium”, “unidentified”). The genus table was further curated to include only data from high-responder mice collected from days 10, 11, and 12. Any genus with 1000 or fewer counts from the subsequent table was also removed from further analysis. Spearman correlation between genera was performed with the Hmisc package (v.4.7–1) in R version 4.1.3. Significant *(P <* 0.05) positive interactions from the resulting correlation matrix were retained. Any genera with a positive correlation coefficient with *Salmonella* were further considered. The resulting correlation network was plotted with R base plot function.

### Metagenome assembled genome database

A total of 3667 (n = 160 dereplicated quality) metagenome assembled genomes (MAGs) were used in this study. These MAGs were (i) obtained from the CBAJ-DB (n = 2281) with methods previously described [29], and (ii) were reconstructed from metagenomic sequencing of six additional mice with methods described here.

All metagenomic reads were checked for quality with FastQC (v0.11.9) and trimmed of low quality reads and adapters, and mouse reads were removed using BBDuk (ktrim = r, k = 23, mink = 11, hdist = 1, qtrim = rl, trimq = 20, minlen = 75, maq = 10) from BBTools (v38.89, https://jgi.doe.gov/data-and-tools/bbtools). Metagenomic samples (n = 6) were assembled independently with Megahit (v1.1.1) and the resulting assemblies were filtered to include only contigs ≥2500 bps and binned with Metabat2 (v2.12.1, −verysensitive). Collectively, all assemblies resulted in 1386 MAGs, which were dereplicated with CBAJ-DB using dRep (v2.6.2, −sa 0.99 -comp 50 -con 10), resulting in 160 dereplicated MAGs which are the basis for this paper. The dereplicated MAG set (n = 160, CBAJ-DB v2.1) was used for mapping metagenomic and metatranscriptomic reads. MAG taxonomy was assigned using GTDB-Tk (v.2.1.1, r207). MAG quality was confirmed with CheckM (v.1.1.2). MAGs were mined for 16S rRNA sequences and matched to ASVs using The Microbial Ecosystem Lab software (https://github.com/WrightonLabCSU/join_asvbins). Briefly, join_asvbins workflow searches MAGs for candidate 16S rRNA sequences using MMseqs2 and a reference database (silva-138-99-seqs-515-806) and it uses Barrnap (v0.9) to predict 16S rRNA sequences. The candidate sequences are then compared to amplicon sequenced ASV sequences using MMSeqs2.

**Figure 2:**
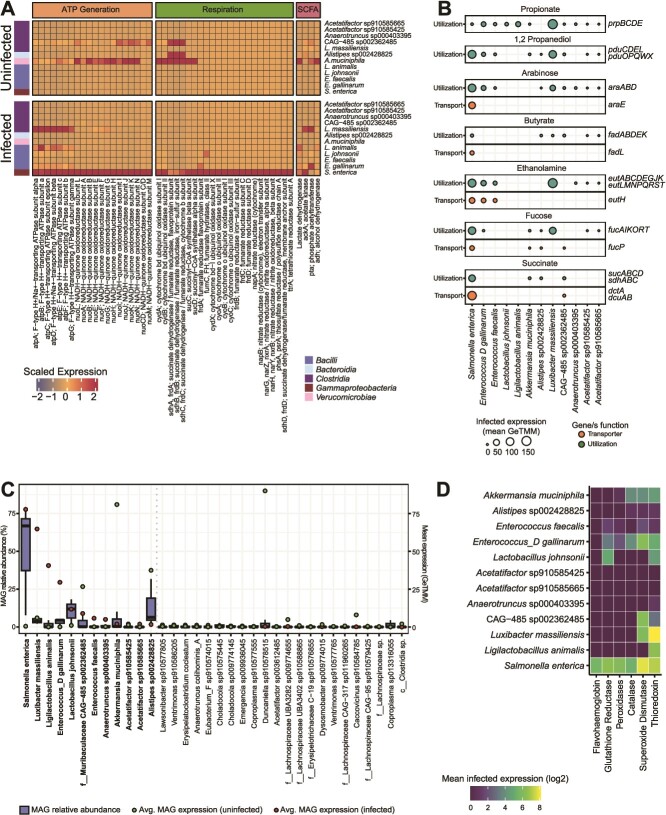
**Microbiota gene expression during infection reveals potential for substrate overlap with *Salmonella.*** (**A**) Heatmap of genome transcript recruitment during infection and there significant differentially expression between treatments. Cell values are GeTMM scaled totals of all genes linked with a particular gene description (x-axis) averaged across samples within a treatment and expressed by an individual taxon (y-axis). Colored boxes on the left indicated gene class. (**B**) carbon utilization and transport expressed by prominent bacteria during *Salmonella* infection. (**C**) box plots (blue) showing the MAG relative abundance distribution (GeTMM normalized mapped reads) of the 35 bacteria with the highest average expression (total annotated genes) in *Salmonella*-infected mice. Red points indicate average MAG expression in the infected treatment and green points indicate average MAG expression in the uninfected treatment. Taxa are ranked left to right by most detected expression during infection. Bold taxa are those with ≥1.5 mean expression (GeTMM) in the infected treatment, a threshold marked by the dotted gray line. (**D**) average expression of oxidative stress response genes by prominent taxa during infection.

Gene annotations for each MAG in the database were produced with DRAM (v1.4.0) [37]. Sulfur metabolism was curated from DRAM annotations, with manual curation of cysteine and serine sulfatase activation signatures*—* (C/S)*XPX*R and CXAXR in R version 4.1.3 [38]. The resulting gene set was further curated to include only genes with a sulfatase motif and Pfam Sulfatase annotation, either sulfatase PF00884.26, or the sulfatase modifying factor enzyme PF03781.19. “Obligate fermenter” designation was assigned if a MAG lacked transcripts of respiratory genes (Fig. 2) and we could find no mention of respiration capabilities in the literature.

### Metagenomic mapping

Raw paired-end metagenomic sequencing reads from *Salmonella*-infected mice used to create the CBAJ-DB v2.1 database and one additional *Salmonella*-infected metagenome (n = 7) were mapped to the CBAJ-DB v2.1 database using Bowtie2 (v.2.4.5) [39]. Prior to mapping, raw reads were quality trimmed with Sickle (v.1.33) to remove sequences with phred quality scores below 20. Additionally, reads were filtered with RQCFilter2 to remove adapter sequences, RNA artifacts, phiX kmers, rRNA sequences, and any sequences that map to mouse, cat, dog, human, or common microbial contaminate genomes. BAM files produced during mapping were sorted by sequence name and filtered to include only mappings with 95% identity with Samtools (v.1.9) and reformat.sh (https://github.com/BioInfoTools/BBMap/blob/master/sh/reformat.sh) respectively. Gene counts were produced for each MAG in the database with Featurecounts (v.1.5.3) and the resulting counts table was GeTMM normalized [40].

### Metatranscriptomic sequencing analysis and mapping

Raw metatranscriptomic paired fastq data collected on day 11 from five CBA/J mice in each treatment were quality trimmed to remove reads shorter than 75 bps and of lower quality than phred 20 with bbduk.sh (https://github.com/BioInfoTools/BBMap/blob/master/sh/bbduk.sh). PhiX and rRNA sequences were removed with bbmap.sh (https://github.com/BioInfoTools/BBMap/blob/master/sh/bbmap.sh) at minid = 0.90. The resulting reads were then mapped to the concatenated quality non-redundant MAG database (CBAJ-DB v1.2) using Bowtie2 (v.2.4.5) using flags -D 10 -R 2 -N 0 -L 22 -i S,0,2.50. Binary alignment files were filtered to only include mappings with high sequence identity (≥97%) using reformat.sh (https://github.com/BioInfoTools/BBMap/blob/master/sh/reformat.sh) and were sorted by sequence name with Samtools (v.1.9). Individual gene counts were produced with Featurecounts (v.1.5.3) and the resulting counts table was GeTMM normalized [40]. The count table was further filtered to remove any genes with 0 counts in every sample. Rarification to 2.5 Gbps was performed with reformat.sh from the BBTools suite on each metatranscriptome and the mapping process was repeated. Active MAG comparison between rarified and unrarified mapping was determined by considering genomes with at least one count in three or more samples.

Differential gene expression and significance of GeTMM normalized filtered transcript counts was produced with limma (v.3.50.3) and edgeR (v.3.36.0) R packages. Limma analysis was performed on all metatranscriptomic samples bar that from mouse I7 which was omitted from the infected group. The DRAM distillate and product outputs were used to group counts assigned to individual MAGs into functional categories and to determine MAG substrate utilization and individual gene expression in MAGs of interest. Metabolic gene expression was determined by a total count value greater than 0 of genes with either a Kegg ID or CAZY ID that were also classified in the DRAM metabolism summary sheets: “carbon utilization”, “Energy”, “Organic Nitrogen”, and “carbon utilization (Woodcroft)”.

## Results and discussion

### Microbiome membership is impacted by *Salmonella* infection

Using 16S rRNA gene amplicon sequencing we evaluated the feces from CBA mice infected with *Salmonella* (n = 44 mice) and an uninfected cohort without *Salmonella* (n = 23 mice) (Supplementary Data File S1**,** 16S rRNA data)*.* From a subset of these mice chosen from later timepoints based on feces availability, we report a collection of metabolite and metatranscriptome samples from seven *Salmonella* infected (infected, I1-I11) and seven non-*Salmonella* infected (uninfected, U1-U8) mice (Fig. 1A). With 16S rRNA gene amplicon sequencing performed at days −2, −1, and 0, as well as days 10, 11, and 12 post-infection we verified later stage infection communities on days 10, 11 and 12 were not statistically different from one another and were distinct from the pre-infection and uninfected communities (Fig. 1B).

We recognize the potential for confounding effects from mouse source material, diet, cage density and coprophagy on the microbiome. To the best of our ability, we strove to mitigate these impacts with our experimental design (see methods). For example, we attempted to overcome cage effect and mouse source material by selecting mice from multiple cages across various experimental rounds. We show consistency with our reported microbiome here and two prior studies using the CBA model [29, 30]. Specifically, *Lactobacillus* and *Enterococcus* were the predominant *Bacilli* enriched with *Salmonella* (Fig. 1C) [29]. In fact, these two members were the only genera that showed positive significant correlations with *Salmonella* at later timepoints of severely infected mice (*Salmonella* relative abundance ≥25% in at least one timepoint) (Fig. S1A). Genera (n = 19) with significant positive correlations to at least one other taxa during infection include *Akkermansia*, *Alistipes*, 14 *Clostridia* genera (Fig. S1A). These results paired with the discriminant analysis results (Fig. 1C) indicate a strong association of *Lactobacillus* and *Enterococcus* with *Salmonella* during later stages of infection and provide evidence of a distinct bacterial community associated with *Salmonella* infection.

Since the earlier publication of CBAJ-DB (v1.0) [29], we have updated the collection to v1.2, by adding MAGs reconstructed from six new metagenomes from CBA mouse feces that were either infected or uninfected with *Salmonella*, resulting in 47 new MAGs in the non-redundant medium and high quality database (contamination <10% and completeness ≥50%) (Fig. S1B, Supplementary Data File S2, MAG data). The database is publicly available (Doi: 10.5281/zenodo.8395759) and includes 3667 total MAGs clustering at 99% genome identity to 160 non-redundant, quality MAGs. We confirmed CBAJ-DB v1.2 included representation of *Enterococcus* (n = 5 MAGs) and *Lactobacillus* (n = 6 MAGs) recovered during infection. We note that the amplicons detected here had perfect matches to MAG recovered 16S rRNA genes from the highest quality Lactobacillus johnsonii MAG (**Supplementary Data S2**), indicating the co-occurring strains during *Salmonella* late infection were well represented in our genomic catalog.

The 10 metatranscriptomes sampled on day 11 from uninfected and *Salmonella*-infected mice were mapped to the CBAJ-DB v1.2 (Fig. S1B). Metatranscriptomes were sequenced to a max depth of 11.4 Gbps and to a mean depth of 6.8 Gbps (mean 5,689 578 paired 151 bp reads), ensuring read recruitment to non-*Salmonella* and lower abundance members of the microbial community (Supplementary Data File S3, metatranscript data). When we rarified the data to 2.5 Gbps, a common value in gut microbiome analyses, it decreased our MAG representation in the infected metatranscriptome by 51%, indicating the value of our deeper sequencing (Supplementary Data File S3, see methods). Seven metagenomes from infected mice sampled on day 8 or day 11 were also mapped to the CBAJ-DB v1.2 (Supplementary Data File S2) to ascertain genome relative abundance in the infected community (Figs. 1A, S1B). The mapped metagenomes were deeply sequenced to a max depth of 68.4 Gbps and a mean depth of 27.4 Gbps (mean 19,655 610 paired 251 bp reads) to avoid sequence saturation from high abundant *Salmonella* and resulting in the recovery of a combined 160 medium and high-quality MAGs all with detectible read mapping from infected metagenomes (Fig. S1B). Due to limited fecal sample amounts at the later days in the experiment, untargeted metabolites were collected on day 12 from each of the 14 mice and metatranscriptomes were collected on day 11 from five mice in each treatment (Supplementary Data File S4, metabolite data). We also report, lipocalin-2, an indicator of enteric inflammation in mice, was measured on mice from each treatment group prior to infection and on the infected group after infection (Supplementary Data File S1, Fig. S2).

### Salmonella expresses genes for diverse carbon and energy metabolic strategies

This *in vivo* metatranscriptomic analysis of *Salmonella* with an otherwise unperturbed microbiota (Fig. 2A) offers insights into genome-resolved strains that recruit metatranscript data during late-stage infection when *Salmonella* relative abundance exceeded 50% (Fig. 1B, S3). On average the *Salmonella* genome recruited 20% of the community metatranscriptome reads from the five infected metatranscriptomes from day 11 (Supplementary Data File S3). It is well documented that *Salmonella* initiates host inflammation that creates oxidized electron acceptors that the pathogen then uses to respire and outcompete the normal flora (Supplementary Table 1) [9, 12, 13]. It is thought that the respiration provides an energetic advantage over the obligatory fermentative commensal members of the gut microbiota [41]. However, it was not known which of these *Salmonella* respiratory metabolisms were expressed during late stages of infection, and if other members of the community also used similar metabolic strategies.

*Salmonella* respiratory processes were evidenced in our transcript data at day 11, when we found indication of aerobic respiration via expression of high and low affinity oxidases (*cydAB*, *cyoABC*, Fig. 2A). *Salmonella* also expressed genes for sulfur respiration, including the genes for tetrathionate (*ttrA*, *ttrB*, and *ttrC)* and thiosulfate reduction (*phsA*, *phsB*, and *phsC*). These oxidized inorganic sulfur compounds can be created by reactive oxygen species (ROS) oxidization of hydrogen sulfide (H_2_S) produced by host colonocytes during inflammation, a process instigated by *Salmonella* [42–45]. Prior reports describe *Salmonella* ability to utilize nitrate, nitrite, and trimethylamine oxidase (TMAO) [46–48] and these metabolisms (*napAB*, *narGHZY*, *nxrAB, torAC*) were also expressed in our data (Fig. 2A, Supplementary Data File S3). We note genes (*torA, torC, torR*) in the gene complex for TMAO reduction had detectable transcripts, but recruitment was much lower than genes to use oxygen, sulfur, or inorganic nitrogen compounds (Supplementary Data File S3). The metatranscript data recruited to the *Salmonella* genome indicates the pathogen expresses a capacity to respire oxygen, sulfur, and nitrogen. We consider it is possible these genes are all co-expressed simultaneously, or that measurement of fecal signature may have aggregated subpopulations located in microsites across the gastrointestinal track. Future studies examining the metabolism within differential regions (mucosal, lumen) within the cecum and other parts of the gastrointestinal track could be warranted to discern the fecal metatranscript bulk signal. Regardless, our findings support the notion that within a complex, intact microbiome, respiration provides an energetic advantage to outcompete commensal fermentative bacteria during infection [9, 41, 45, 46, 49], which all had much lower respiratory metabolism transcript levels (Fig. 2A).

We also note that there was within mouse heterogeneity in *Salmonella* responses. For example, relative to the other mice, mouse I7 was less colonized by *Salmonella* at the time of metatranscript sampling (Fig. 1B). Concomitantly, this mouse displayed less recruitment of respiration related genes, with *Salmonella* transcript levels for nitrate reduction (*narL*) and thiosulfate reduction (*phsC)* being 4 times and 47.5 times lower respectively in the I7 mouse compared to the other infected mice (Supplementary Data File S3). This mouse-to-mouse variation is worth noting, as extending these approaches to time series, could mean that normalization to inflammation intensity or *Salmonella* abundance may better inform infection responses rather than time since inoculation.

We next explored *Salmonella* carbon utilization as potential substrates for competition with commensal gut membership. It has been proposed that this respiratory capacity allows *Salmonella* to use lower energy compounds unavailable to many obligate fermenters, such as propionate, succinate, 1,2 propanediol, and ethanolamine as well as utilize sugars like fucose and arabinose [8, 12, 15, 50–55]. During day 11 of infection *Salmonella* expressed genes for the utilization of all the listed carbon sources, and the operons for arabinose (*ara*), 1,2 propanediol (*pdu)*, and ethanolamine (*eut*) were the most highly transcribed of the carbon utilization genes examined (Fig. 2B, Supplementary Data File S3). We observed overlap in carbon use gene expression by some commensal bacteria in infected microbiomes, indicating a potential substrate-use overlap with *Salmonella* that could be tunable for enhanced metabolic competition in future probiotic interventions (Figs. 2B, S2).

### Diverse microbiota transcribe metabolic genes during *Salmonella* infection

We were next interested in the genome-resolved transcription of non-*Salmonella* microorganisms persisting during *Salmonella* infection. *Salmonella* was the most dominant bacterium in the infected samples (50.3% average genome relative abundance, Fig. S3) followed by *L. johnsonii* (15% average relative abundance) (Figs. 2C, S3, Supplementary Data File S1). *Salmonella* also displayed the highest metatranscript recruitment during infection (77.7 mean GeTMM), *Luxibacter massiliensis* (*Clostridia*)*, Ligilactobacillus (*formerly *Lactobacillus B*) *animalis* (*Bacilli*), and *Enterococcus D gallinarum* (*Bacilli*), and *L. johnsonii* (*Bacilli*) were the next lineages in infected fecal communities with the most transcription detected (Figs. 2C, S3). This response reflected the *Bacilli* class enrichment reported in our 16S rRNA analyses (Fig. 1B).

Highlighting the discrepancy between genome abundance and transcript recruitment in the feces, *Alistipes* sp002428825 (*Bacteroidia*) and Akkermansia muciniphila (*Verrucomicrobiae*) were some of most dominant genomes of the infected gut (3^rd^ and 4^th^ ranked in the community by genomic DNA), yet their average transcription during infection was only 1.67 and 2.03 mean GeTMM respectively (10^th^ and 11^th^ ranked in the community by RNA) (Fig. S3). We consider this difference between expression and genome abundance in the community could because *Alistipes* and *Akkermansia* were still present in the microbiome, thus not impacting the relative abundance in genome content, but were expressing less genes in response to infection induced chemical changes. Alternatively, we note the data used in this study was fecal material, not cecum and that transcriptional responses may be altered from the cecum signal, a consideration especially important for *Akkermansia* and other mucus layer associated taxa [30]. Since our prior published work using 16S rRNA gene amplicon sequencing revealed no statistical differences in late infection cecum and fecal communities in terms of microbiota membership and relative abundance [30], and feces are often used as a proxy for microbial insights in humans and mice, fecal samples were used to support this multi-omics investigation.

We found evidence that 56 MAGs other than *Salmonella* transcribed metabolic genes during infection (Supplementary Data File S3), a phylogenetically diverse group of genomes belonging to phyla *Actinobacteria* (n = 1), *Verrucomicrobiota* (n = 1), *Bacteroidota* (n = 4), *Firmicutes* (n = 5), and *Firmicutes A* (n = 45). The most enriched (≥ 1.5 mean GeTMM mapped transcripts) members in feces from the *Salmonella* infected gut included 11 members of the *Lactobacillaceae*, *Lachnospiracea*, *Enterococcaceae*, *Muribaculaceae*, *Ruminococcaceae*, *Rikenellaceae*, and *Akkermansiaceae* (Fig. 2C). Consistent with our previous report, we showed interesting complexity in the *Clostridia* with some genomes maintaining high relative abundance during infection while other dominant genera became reduced during infection [29]. We note four of the top 11 most transcriptionally enriched lineages in infected mice from this study are of the *Clostridia* class (Fig. 2C) and 45 *Clostridia* MAGs transcribed metabolism related genes in infected metatranscriptomes (Supplementary Data File S3). In total 52 bacterial lineages, including the most transcriptionally enriched bacteria, co-expressed some of the same genes for carbon and energy utilization as *Salmonella*, indicating community-wide metabolic overlap and potential exchange were occurring at this stage of infection (Figs. 2A, S3, S4). Of the top 11 most transcriptionally enriched infection MAGs, we detected more transcripts from eight in infected mice than in uninfected mice (Fig. S3), showing how *Salmonella* infection enriches for new transcriptionally active community members.

### Genome-resolved, differential transcription reveals changes between the inflamed and non-inflamed gut

It is well reported in the *Salmonella* literature, that infection increases inflammation, which leads to increased reactive oxygen, nitrogen, and sulfur species that allow for pathogen proliferation using respiration to outcompete the obligate fermenters in the gut [49–51]. Here we use lipocalin as marker of enteric inflammation, showing that that *Salmonella* infection increases inflammation significantly from prior to infection levels (Fig. S2). New to our study, is the impact that these chemical changes caused by pathogen-induced inflammation may have on the rest of the microbiome in terms of transcript responsiveness. To determine how the key infection detected genomes retooled their transcribed metabolisms from non-inflamed to inflamed infection conditions we performed a differential transcript analysis at the MAG level across the two treatments.

Consistent with the need to withstand oxidative conditions that allow Salmonella to respire (Fig. 2A), *A. muciniphila* and *Muribaculaceae* CAG-485 sp002362485 transcribed genes for reducing oxygen under microaerophilic or lower oxygen conditions (*cydAB*), albeit with less transcripts recruited to these MAGs in the infected than the control (Fig. S5). Perhaps this is because of competition for molecular oxygen with *Salmonella* which transcribed both low (*cyoABC*) and high affinity oxidases (*cydABX*) (Fig. 2A), or lower overall all genome wide transcript recruitment*.* We consider oxygen respiration could support growth, as *Akkermansia* has been reported to use NADH dehydrogenase in concert with cytochrome bd ubiquinol oxidase to respire in microaerophilic conditions [56] and both were co-transcribed under infection (Fig. 2A, S5, Supplementary Data File S3). Although the literature is scant concerning *Muribaculaceae*, our data suggest growth with oxygen respiration may be possible as the organism had transcripts for NADH dehydrogenase and oxidases co-detected during infection (Fig. 2A, Supplementary Data File S3) [57]. Thus, it is possible that these organisms may have some encoded and transcribed mechanisms to withstand and may even grow respiratorily in the inflammatory environment created by *Salmonella* infection.

Given the breadth of respiratory metabolism expressed by *Salmonella*, we were interested if other dominant and persisting members in the infected gut utilized a variety of respiratory energetic strategies. Others have shown that *Salmonella* inflammation increases the availability of nitrate and tetrathionate (Supplementary Table 1), which was thought to favor *Salmonella* expansion in the gut over other organisms that lacked this capacity [9, 12, 45, 58]. However, these experiments were not tested in a complex, non- augmented microbiome. Analysis of the infection-enriched taxa failed to find any transcriptional evidence for denitrification or sulfur respiration pathways. Taken together, our findings from more diverse community backgrounds, suggest the use of oxygen may support respiration by other infection microbiota, but that *Salmonella* uniquely responds to the presence of anoxic electron accepting compounds. Acknowledging there are constraints to multi-omics data interpretation, including but not limited to inaccurate functional annotations and that transcriptional may fail to manifest as enzyme activity, future physiological studies are warranted. These could include competition studies with isolates of these strains paired to measured growth and electron acceptor use to support these multi-omic suppositions.

Gene transcripts for fumarate reduction by *Enterococcus D gallinarum* and *L. johnsonii* increased between infected and non-infected conditions (Fig. 2A, S4, S5). Based on genomic content of this ~96% complete *Enterococcus* genome, that lacked electron transport chain, terminal oxidase, and complete tricarboxylic acid, we consider this organism likely to be an obligate fermenter and is thus not respiring fumarate. As shown for other lactic acid bacteria (Lactobacillus, Enterococcus), fumarate could provide a growth advantage not for respiration, but as hydrogen sink during sugar fermentation [59].

We considered it likely that like *Salmonella* [60] and the persistent commensal bacteria employed mechanisms to tolerate reactive oxidative stress conditions during infection (Fig. S3, Supplementary Data File S3). The most common transcribed strategy across the infected community was thioredoxin, where thioredoxin-dependent peroxiredoxin (*tpx*) reduced ROS in a manner dependent on thioredoxin 1 (*trxA*) [61]. We note that peroxidases, thioredoxin and superoxide dismutase were not significantly differentially expressed between the infected and uninfected conditions (3X or less enrichment in either treatment). Alternatively, catalase was more transcribed in the uninfected gut (14 times more enriched), and flavohaem and glutathione reductase (*gor*) were 48 and 248 times more transcriptionally enriched in the inflamed gut (Fig. 2D). The latter, *gor,* is important to lactic acid bacteria like *Enterococcus*, *Lactobacillus* and *Liglactobacillus* for maintaining reduced glutathione amidst oxidant stress from host immunity [62].

In summary, the ability to respire or withstand oxidative stress appears transcriptionally enriched in genomes that persist and transcribe genes in the inflamed, *Salmonella* infected gut. This multi-omics research outlines new areas for data collection in mouse models through measurements of these electron acceptor compounds during infection in both gnotobiotic (host only), as well as gnotobiotic with *Salmonella*, and the intact microbiome with *Salmonella*. We note like our study, these compounds are often inferred in earlier studies and often not directly measured. We also acknowledge the need for physiological co-culture studies between isolated near-neighbors to these infection resistant strains and *Salmonella* to verify possible respiratory competition with *Salmonella*, as well as other avenues for growth advantages, e.g. via fumarate reduction by infection-resistant lactic acid bacteria.

### Intertwined lactate metabolisms are expressed during *Salmonella* infection

**Figure 3:**
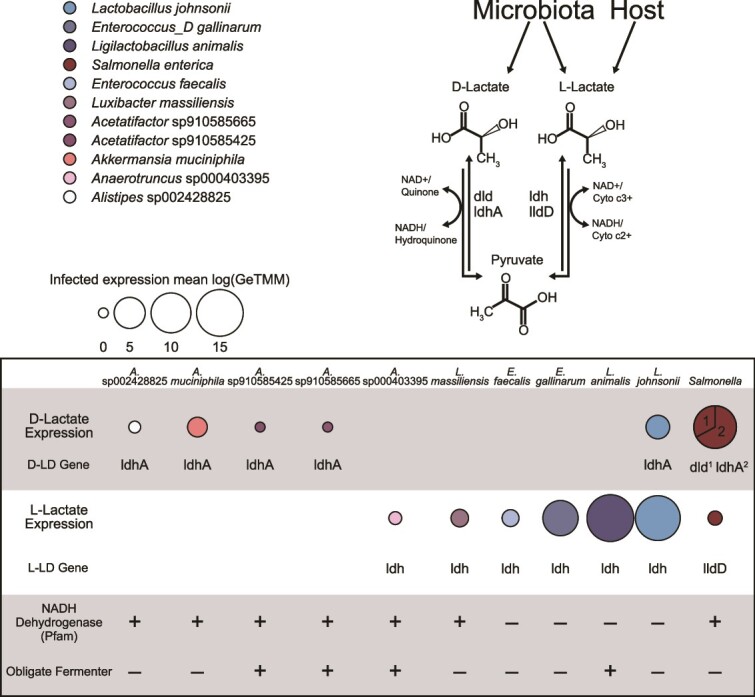
**Co-expression patterns indicate lactate cross-feeding and competition during infection.** Expression of individual lactate dehydrogenase genes by select taxa during *Salmonella* infection. Circle sizes indicate average log expression in infected samples recruited by individual genomes or averaged within higher taxonomic groups. Numbered sections in the *Salmonella* pie chart denote the proportion of total lactate dehydrogenase expression represented by each gene. NADH dehydrogenase gene content was examined for each MAG, a plus (+) indicates at least one Pfam annotated gene is present, a minus (−) indicates no genes were annotated “NADH dehydrogenase”. Positive (+) obligate fermenter designation was assigned if a MAG showed no expression of any respiratory genes as surveyed in Fig. 2. Negative (−) obligate fermenter designation was applied to all other MAGs in the figure and is supported by literature discussed in the text.

Our metatranscript analysis indicated lactate was a possible carbon source used by *Salmonella* (Figs. 2A, S4). This metabolite was likely important community-wide, with 11 of the most transcriptionally active genomes in the infected intestine expressing lactate dehydrogenase genes (Fig. 3). In support of this, we detected the lactate metabolite in 3.9-fold greater mean abundance in the infected mice than in the non-infected mice (Supplementary Data File S4).

Studies using *Salmonella* genetics in gnotobiotic or reduced complexity microbiomes reported that L-lactate from colonocytes was the primary lactate enantiomer used by *Salmonella* [7, 63]. Our metatranscriptome data offered an opportunity to track D- and L-lactate use by gene expression, via lactate dehydrogenase gene specificity for different lactate enantiomers – *ldh* (L-lactate dehydrogenase, K00016), *lldD* (L-lactate dehydrogenase, K00101), *dld* (D-lactate dehydrogenase, K03777), and *ldhA* (D-lactate dehydrogenase, K03778). We note caution in inferring the sources and sinks of lactate from gene transcript data alone, as many microorganisms like LAB (e.g. *Lactobacillus* and *Enterococcus* genera) produce lactate when fermenting, and bacteria like *Clostridia* can ferment lactate to butyrate or acetate, and *Salmonella* can oxidize lactate when respiring [64]. Here the directionality of lactate use or production was inferred by gene type, energy favorability, and defined capabilities of closely related characterized strains [7, 63, 64]. Of note however, these suppositions are made solely based on metatranscriptomic data which do not necessarily indicate enzyme production or substrate consumption leading to growth, and thus would need validation using physiology-based experimentation.

*Salmonella* transcribed genes for both lactate enantiomers, with genes for D-lactate exceeding that for L-lactate (Fig. 3). *Salmonella* was also the only organism with detectable transcripts for both lactate dehydrogenase genes for D-lactate (*dld*, *ldhA*, Fig. 3). Given its respiratory capacity and its known use of lactate as a substrate [65], *Salmonella* is plausibly consuming D-lactate. Though many factors may influence gene transcription, these findings show D-lactate possibly produced by the microbiota, and L-lactate previously thought to be colonocyte-derived, are both possible substrates used by *Salmonella* in an intact microbiome.

We next examined if microbial community transcription supported a model where D-lactate was produced by members of the microbiota. Of the most prominent infection community members, *L. johnsonii, Alistipes* sp002428825, *A. muciniphila*, and two *Clostridia* had detectable levels of D-lactate dehydrogenase (*ldhA*) transcription in infected samples (Fig. 3). Outside of *Salmonella*, *ldhA* transcription was highest from *L. johnsonii*, displaying a significant 3.48 log fold-change increase of *ldhA* transcription during infection, likely due to its nearly 38-fold enrichment under the same conditions (Fig. 3, S5). Of the D-lactate metabolizing strains, physiological characterization of isolates closely related to this MAG demonstrated that *L. johnsonii* strains produce both D and L-lactate [66], often times in a 60/40 (D/L) racemic proportion [66, 67], supporting the notion that lactate was likely produced [68]. Prior work deduced *Salmonella* preference for host derived L-lactate in gnotobiotic mice [7]. We posit *Salmonella* could alter lactate utilization methods based on competition with the microbial community and greater the D/L enantiomer proportion from LAB activity in the infected microbiome.

Most of the L-lactate dehydrogenase transcription occurred in *Bacilli*, especially LAB like *Enterococcus spp*., *L. johnsonii* and *Ligilactobacillus animalis*, which are known to produce L-lactate [64, 66, 69]. We show this production is important during infection, where L-lactate dehydrogenase (K00016) was upregulated 1.8 and 3.9 mean log fold-change respectively in the infected treatment (Fig. 3, S4). MAGs closely related to *Enterococcus* species *gallinarum* and *faecalis* also highly upregulated L-lactate dehydrogenase (K00016) during infection 4.3 and 3.8 mean log fold-change (Fig. 3, S4). Taken together we consider it likely that in addition to colonocytes, the microbiota are another source of L-lactate during infection. Alternatively, although LAB may produce L-lactate, we cannot rule out that other fermentative members like *Clostridia* (e.g. *Luxibacter, Acetatifactor* spp., and *Anaerotruncus*) may instead ferment lactate, as demonstrated by other members of this class [64], resulting in low net L-lactate production. These results, along with the *Salmonella* data, point to lactate cross feeding as potentially important for persistence during inflammation.

Combined our metabolite and genome-resolved metatranscript data expands the possible fates of lactate metabolism in the infected microbiome. Taking our findings in light of the existing framework, we consider it possible that both host-derived and microbiota-derived (specifically from transcriptionally active LAB) lactate is an important metabolite in the infected gut. Besides production, the transcript data suggests *Salmonella* can utilize both lactate enantiomers, but also that clostridial strains could compete with *Salmonella* for lactate in the inflamed gut. Moving beyond metabolite and transcript insights alone, studies using isotopically labeled carbon sources fed to mice with may help detangle the sources, as well as cross feeding of key carbon metabolites in the gut.

### Metabolites from the infected microbiome indicate a likely prevalence of organic sulfur pools in the gut

**Figure 4:**
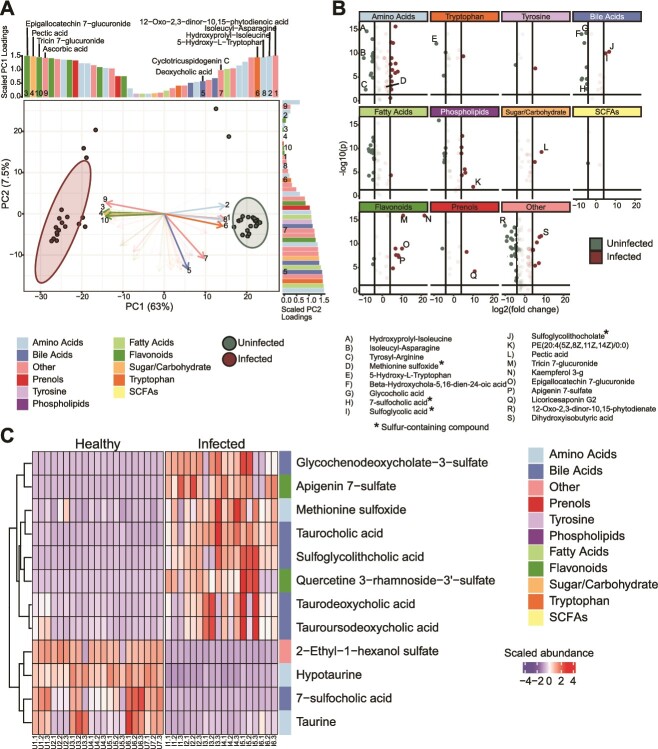
**Uninfected differ from inflamed metabolomes in sulfonated bile acids and flavonoids.** (**A**) principal component analysis (PCA) of metabolites from infected and uninfected mice colored by treatment (red = infected, green = uninfected). Arrows and bars are significant (ANOVA *P <* 0.05) compounds. Dark arrows are the top 10 compounds that explain the PCA ordination variance (Euclidian distance from tip to centroid). Bars are arranged by absolute value of compound loading for each component, and they are colored by compound group. (**B**) volcano plots of significant metabolites separated by compound group. Points are colored by treatment (red = infected, green = uninfected) and dark points indicate significant (ANOVA *P <* 0.05) compounds with at least 4x log2 fold change between treatments. Select compounds (most changed or system relevant) are labeled below the plot and sulfur containing compounds are denoted with an asterisk. (**C**) Heatmap showing the center scaled abundance of significantly different sulfur containing compounds in each treatment.

Untargeted metabolomics compared the metabolomes in uninfected mice and those at the later stages of infection. Broadly, we noticed a significant (PERMANOVA, *P* value = 0.001) difference in chemical composition of feces from mice 12 days post infection and feces from uninfected, control mice collected at the same timepoint (Supplementary Data File S4). Principal component analysis revealed strong clustering of metabolomes by treatment due largely to differences in abundance of flavonoids, amino acids, and bile acids (Fig. 4A). We found that many sulfonated compounds including sulfated flavonoids, bile acids, and sulfur containing amino acids like taurine and methionine differed in abundance between treatments (**Figs. 4AB**). Sulfonated bile acids were significantly (ANOVA, *P* value<0.05) more abundant in infected guts, as were oxidized methionine and sulfonated flavonoid species, and uninfected mice harbored higher abundances of deconjugated bile acids and free taurine (Fig. 4C).

**Figure 5:**
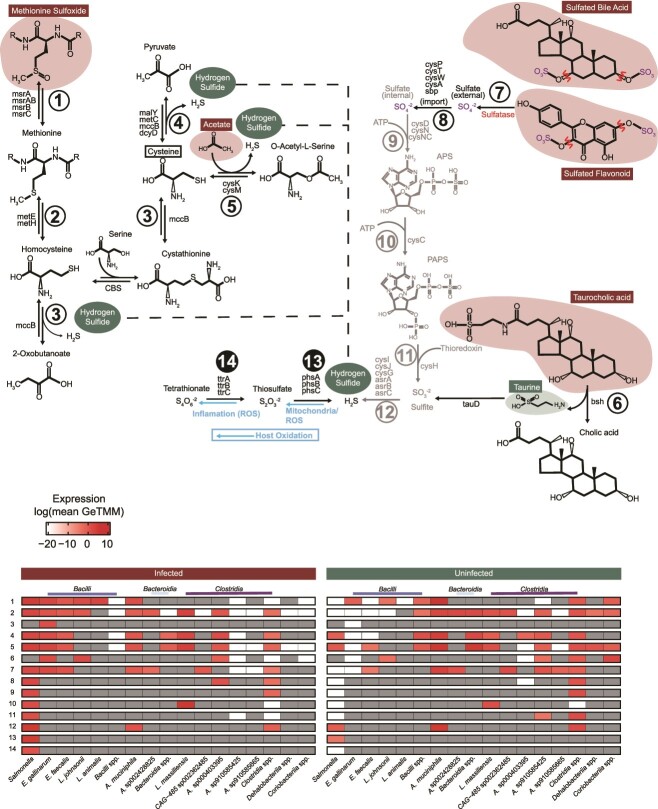
**Community expression profiles implicate commensal membership supports *Salmonella* sulfur metabolism during infection.** Pathways depicting amino acid (left) and sulfated bile/flavonoid compounds (right) as organic sulfur substrates used by the infected microbial community to produce hydrogen sulfide. Numbered pathway arrows correspond with numbered rows in the heatmaps. Dashed lines connect sections of the amino acid pathway to the hydrogen sulfide pool. Blue arrows show host oxidation of inorganic sulfur. Sulfate (purple) released by sulfatase activity (indicated in red) precedes sulfate reduction steps following ABC transporter-mediated translocation colored gray. Compounds outlined in red, and green are significantly more abundant in infected metabolomes or uninfected metabolomes respectively, and compounds outlined in black are not present in our metabolite data. The heatmap on the right indicates pathway expression in uninfected mice and the heatmap on the left indicates expression from infected mice. Cells are colored by average log expression and are dark grey if no expression was detected. Pathway steps denoted with black filled circles are specific to *Salmonella* in the community.

The organic sulfur pool of sulfated bile acids, sulfated flavonoids, and methionine sulfoxide in infected mice provides potential sources of inorganic sulfur via commensal and *Salmonella* sulfatase activity (Figs. 4C, 5). Host-derived sources of sulfur, flavonoids and bile acids, are conjugated to sulfonate in the liver and released back to the gastrointestinal tract via enterohepatic circulation [70, 71]. One of the most discriminant sulfur compounds in our metabolome was methionine sulfoxide, the oxidized form of methionine, potentially originating from dietary protein or dead microbial cells [72]. One report describes the importance of methionine to *Salmonella* virulence, remarking how significant it is that *Salmonella* encodes redundant mechanisms for its acquisition or production [73], and so we chose to examine pathways for utilization of this amino acid more closely in our data.

### Oxidized inorganic sulfur made available by the gut microbiota supports *Salmonella* sulfur respiration

Our data revealed methionine sulfoxide was a key discriminant metabolite prevalent in infected microbiomes with *Salmonella*. We hypothesized this organic sulfur source and its derivatives, could facilitate sulfur exchanges that ultimately lead to *Salmonella* respiration. Given that reactive oxygen species can abiotically oxidize H_2_S yielding *Salmonella* respiratory compounds tetrathionate and thiosulfate (Supplementary Table 1), we were interested in the steps to generate these reduced sulfur pools of H_2_S from organic sources like methionine sulfoxide. Outlined (Fig. 5) is the initial conversion of methionine sulfoxide to methionine (step 1), then to homocysteine (step 2), ultimately yielding 2-oxobutanoate, pyruvate, or O-acetyl-L-serine and H_2_S (steps 3, 4, and 5). Many members of the inflamed gut transcribed the first two steps in this pathway—step 1 methionine sulfoxide reductase (*msrABC*) and step 2 homocysteine methyltransferases (*metEH*). *Salmonella*, *Akkermansia*, and most predominant *Bacilli* actively transcribed these genes, showing methionine sulfoxide is potentially an important sulfur source in the inflamed gut. However, only *Enterococcus D gallinarum* encoded and transcribed genes for cystathionine-γ-lyase (*mccB*) to convert homocysteine to 2-oxobutanoate and cysteine to pyruvate, reactions that release H_2_S and were only transcribed in the infected gut [74].

Genes involved in cysteine metabolism *(*step 4: *dcyD*, *malY*, *metC*, and *mccB*), as well as cysteine synthase (step 5: *cysK*, *cysM*) were examined, as these steps could release H_2_S from cysteine degradation [74]. These pathways were more prevalently expressed than the complete methionine pathway (Fig. 5). *Salmonella*, *Akkermansia*, *Enterococcus*, and *Clostridia* including *Luxibacter* and *Anaerotruncus* expressed both steps 4 and 5 at high levels to release H_2_S during infection (Fig. 5). In summary, our paired genome-resolved transcript and metabolite data provide evidence that sulfur-containing amino acids like cysteine and methionine can be microbially converted to H_2_S, suggesting a role of microbiota metabolism in assisting *Salmonella* sulfur cycling in the inflamed microbiome.

Additional organic sulfur compounds prevalent in our infection data and discriminate between treatments were sulfated bile acids (e.g. Sulfoglycolithocholate, Glycochenodeoxycholate-3-sulfate) and flavonoids (e.g. Quercetin 3-rhamnoside-3′-sulfate, Apigenin 7-sulfate). It is recognized that *Salmonella* and other gut microbes express sulfatases that desulfurize these compounds releasing sulfate (Fig. 5) [75]. Sulfatase transcription by the 12 most active bacteria (excluding *Akkermansia*) was 2-fold greater in the inflamed gut, and Enterococcus faecalis*, Enterococcus D gallinarum,* and *Salmonella* accounted for 94% of all sulfatase transcription in the infected microbiome (Fig. 5). *Akkermansia* sulfatase transcription dramatically decreased in the infection treatment (45.8-fold) (Fig. 5). Overall, these data contribute to a growing body of work aimed to better understand bacterial production of sulfatase in the mammalian colon and its implication on microbiome structure and nutrient acquisition from host-derived and diet-derived sources [76, 77]. Our transcript data emphasizes *Enterococcus* as a likely culprit in transforming the gut organic sulfur pool generating a source of free sulfate during *Salmonella* infection.

Reduction of sulfate (generated from sulfatases) could be a secondary pathway to the production of H_2_S. Although not measured in our study, in the mammalian gut up to 18.6 mmol of sulfate per day is estimated to be reduced, generated from organic sulfur sources like bile acids and mucins [74]. Sulfate is reduced in the gut either via the dissimilatory sulfur reduction (DSR) pathway (*sat, aprAB, dsrAB*) or the assimilatory sulfur reduction (ASR) pathway (*cysDNCHIJ, asrABC*) [74, 78–80]. We detected no DSR gene content in any of the genomes in CBAJ-DB v1.2. Instead, we found genes for ASR transcribed by both *Akkermansia* and *Salmonella* (step 12, Fig. 5), but only *Salmonella* contained genes in the four other steps for importing and converting extracellular sulfate to sulfite, all of which were highly transcribed during infection (Fig. 5). Additionally, three *Clostridia* lineages expressed either *asrA, asrB,* or *asrC* to catalyze the same reduction (Fig. 5) [74, 79]. Our gene data suggest ASR enzymes may be important contributors to sulfate and sulfite reduction, more so than canonically studied DSR processes. These findings from the infected mouse gut are supported by a study in from the human gut, where microbial ASR genes were twice as abundant as DSR genes and deemed critical modulators in colon cancer [74].

We highlight two distinct mechanisms for H_2_S generation during infection using microbiota organic sulfur conversions, including: (i) amino acid degradation and via (ii) removal of sulfate from sulfated organic compounds like bile salts and subsequent reduction via anaerobic sulfite reductase. This reduced sulfur can be abiotically oxidized using ROS to generate tetrathionate and thiosulfate, respiratory electron acceptors. In addition, host catalyzed sulfur oxidation could also generate tetrathionate and thiosulfate in the infected gut, as previously reported [9, 18, 42]. Mining of CBAJ-DB v1.2 genomes for these sulfur respiratory capacities revealed only *Salmonella* had capacity for both tetrathionate and thiosulfate reduction (steps 13, 14 in Fig. 5). Two other *Coriobacteriia* lineages had genes to reduce thiosulfate, yet combined, these lineages comprised less than 0.001% of the infected community (Supplementary Data File S1). One other lineage (*Adlercreutzia muris*) besides *Salmonella* transcribed genes for thiosulfate reduction genes during infection, yet with recruitment 50-fold lower than *Salmonella*. Likewise, both tetrathionate and thiosulfate metabolisms were not transcribed in the uninfected gut (Supplementary Data File S3).

Our lactate and sulfur findings underscore the complex interplay between pathogen, diet, microbiota, and host in the murine gut model. Our results are supported by a growing body of research in humans, recognizing the importance of a balanced sulfur cycle to gut health, where imbalances were linked to inflammatory bowel disease and colorectal cancer [74, 81]. Likely, the sulfur metabolisms shown here by metabolite and metatranscriptomic analysis may be amplified in the westernized human diet compared to the mouse. For example, western diet studies using fecal homogenates demonstrate higher production of H_2_S from organic sulfur amino acids compared to inorganic sources, and high protein intake increased the sulfur-free amino acid content of the gut [82, 83].

While the results generated here present a perspective on how inflammation can alter the microbiome structure and function, caution should be employed when extending results from mouse models to humans. Reasons for this include host diet differences, with more human realistic diet regimes needing evaluation in mouse models to more realistically assess diet-microbiota-pathogen linkages. Moreover, because of microbiome membership differences between hosts, it can be challenging to translate specific gut microbiome from murine to human conditions. However, previously we extensively curated our MAGs to strains recovered from the human gut, showing critical genomes discussed here were 99% similar to strains recovered from inflamed humans (e.g. A. muciniphila or *Enterococcus D gallinarum)* [29]. Thus, physiological insights from these members may have more direct human relevance. Despite these complexities, our approach has illuminated significant relationships between inflammation and microbiome dynamics that underscore the potential for translational research, provided the models and methodologies are carefully aligned with human physiological conditions.

## Conclusions

The work presented here identified a set of commensal bacteria persistent during *Salmonella* inflammation and infection that participate in the utilization and production of key metabolites in the inflamed gut ecosystem. These results offer multiple exciting avenues for probiotic identification of strains robust to inflammation and pathogen perturbation that may be pivotal to re-establishing normal gut function following infection. We confirmed *Salmonella* transcription of genes to utilize lactate, tetrathionate, and thiosulfate *in vivo* amidst an intact microbial community. Our data also expands current paradigms of *Salmonella* lactate utilization during infection and proposes a possible role for commensal bacteria in *Salmonella* sulfur and lactate use. Additional research, ideally with isotopically labeled substrates and defined consortia may help confirm multi-omic based assertions made here. The inferences drawn underpin the value in holistic examination of the microbiome afforded by a multi-omics approach, findings that can corroborate and expand assertions deduced from more reduced complexity model systems.

## Supplementary Material

Final_Post_Supplemental_08192024_wrae187

Supplemental_table_1_wrae187

Supplemental_dataS1_FormerS3_16S_wrae187

Supplemental_dataS2_formerS1_MAG_wrae187

Supplemental_dataS3_formerS2_metatranscript_wrae187

Supplemental_dataS4_formerS4_metabolite_wrae187

## Data Availability

Sequencing data supporting the results shown here are provided in the National center of biotechnology Information (NCBI) under Bioproject number PRJNA348350. The amplicon sequencing ASV table is provided in the Supplementary Data File S1 and CBAJ-DB v1.2 MAG fastas are provided on Zenodo https://doi.org/10.5281/zenodo.8395759. Scripts used in data analysis can be found at https://github.com/ileleiwi/Salmonella-Multiomics-Paper.
