# Supplementary material for: Gut microbiota carbon and sulfur metabolisms support Salmonella infections
Source: ISME J. 2024 Oct 15;18(1):wrae187. doi: 10.1093/ismejo/wrae187 (PMC11482014; doi:10.1093/ismejo/wrae187)
Supplement: Final_Post_Supplemental_08192024_wrae187 [file final_post_supplemental_08192024_wrae187.docx]

**Gut microbiota carbon and sulfur metabolisms support *Salmonella* infections**

Ikaia Leleiwi^1,2^, Katherine Kokkinias^2,3^, Yongseok Kim^4^, Maryam Baniasad^4^, Michael Shaffer^2^, Anice Sabag-Daigle^5^, Rebecca A. Daly^2^, Rory M. Flynn^2^, Vicki H. Wysocki^4,6^, Brian M. M. Ahmer^5^, Mikayla A. Borton^2^, Kelly C. Wrighton^1,2,3*^

**Fig S1: *Salmonella* correlation with *Bacilli* genera in later stages of infection and multi-omics sequencing effort. A** Positive significant (p<0.05) Spearman correlations between community genera and *Salmonella*. Edges are the interaction Spearman correlation coefficient. Taxa listed on the right are all genera, and their mean relative abundances (16S rRNA), that displayed at least one significant positive correlation to another bacteria in later stage (days 10-12) infected samples. **B** Metagenomic and metatranscriptomic sequencing depth. Bars with asterisks indicate metagenomes used to produce the CBAJ-DB v1.2.

**
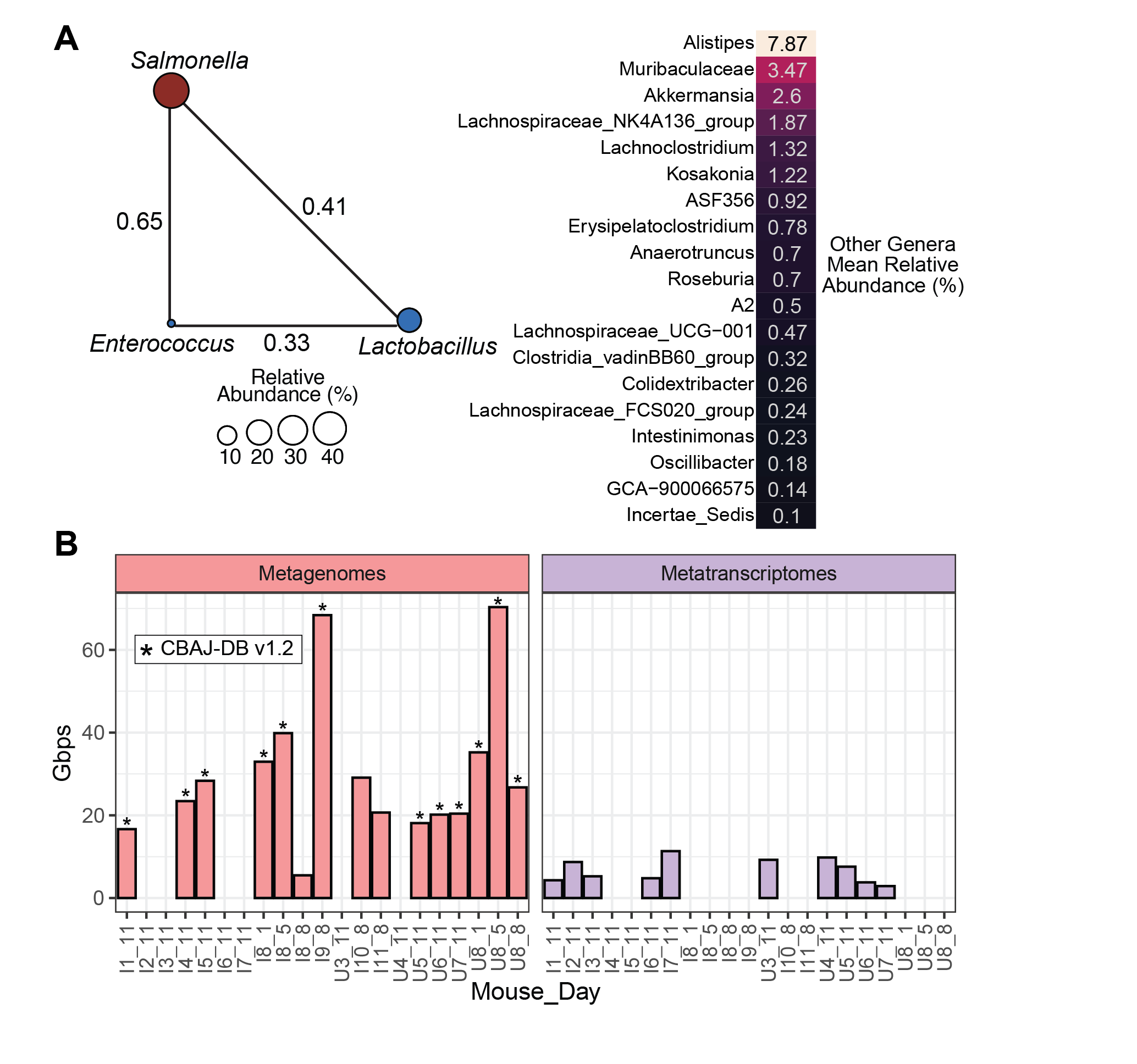
**

**Fig. S2: Lipocalin-2 Levels in CBA/J Mice Both Uninfected and Infected with *Salmonella.*** Lipocalin-2 levels measured from feces taken on either 1 or 2 days pre-infection (n = 9) or 11 and 12 days post infection (n=8). Significance was determined with Dunn’s test of multiple comparisons. No difference was reported in inflammation between the starting communities prior to inoculation with a significant increase in lipocalin post-infection.

**
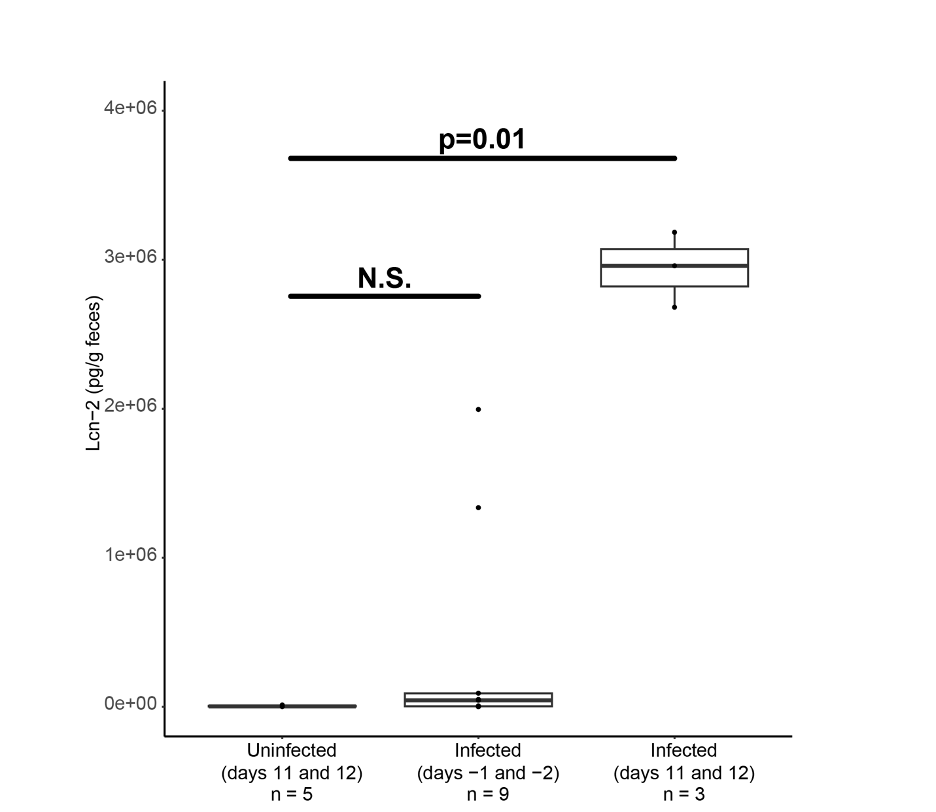
**

**Fig. S3: Low to High Expression Scale for Infected and Uninfected Mice.** Mean normalized expression of mapped transcriptomic reads to the 25 most relatively abundant genomes. Expression is set to a scale from Low to High to add context to instances in the manuscript that refer to either low or high expression levels.

**
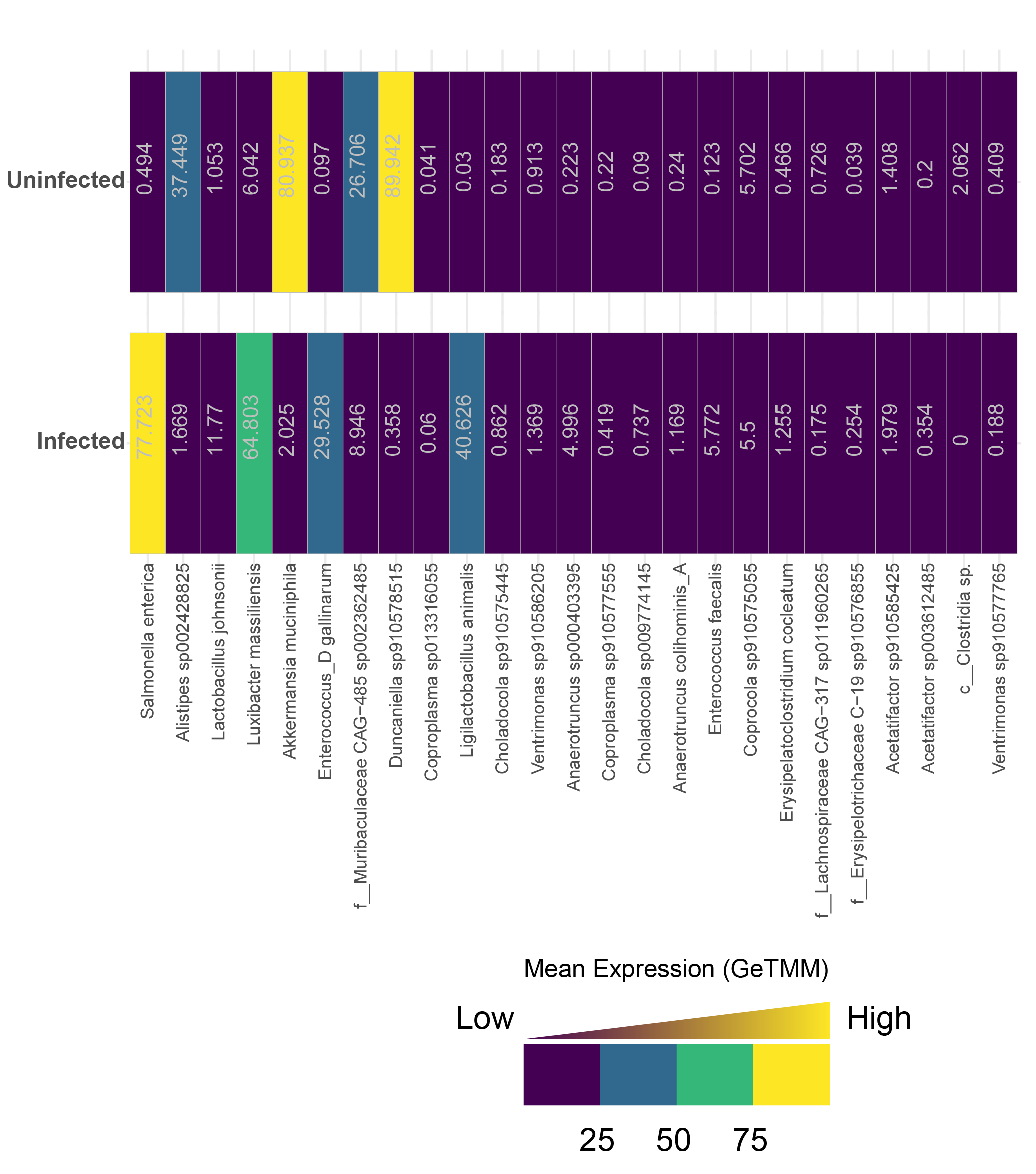
**

**Fig. S4: Carbon utilization of important microbiota in the infected gut.** Carbon utilization of the most active and relatively abundant bacteria in the infected gut as determined by mapped metatranscriptomic reads to individual MAGs. Utilization categories were assigned by DRAM v1.4. Backbone cleavage denotes the presence of an endo-cleaving glycoside hydrolase and oligo cleavage denotes the presence of a exo-cleaving glycoside hydrolase.

**
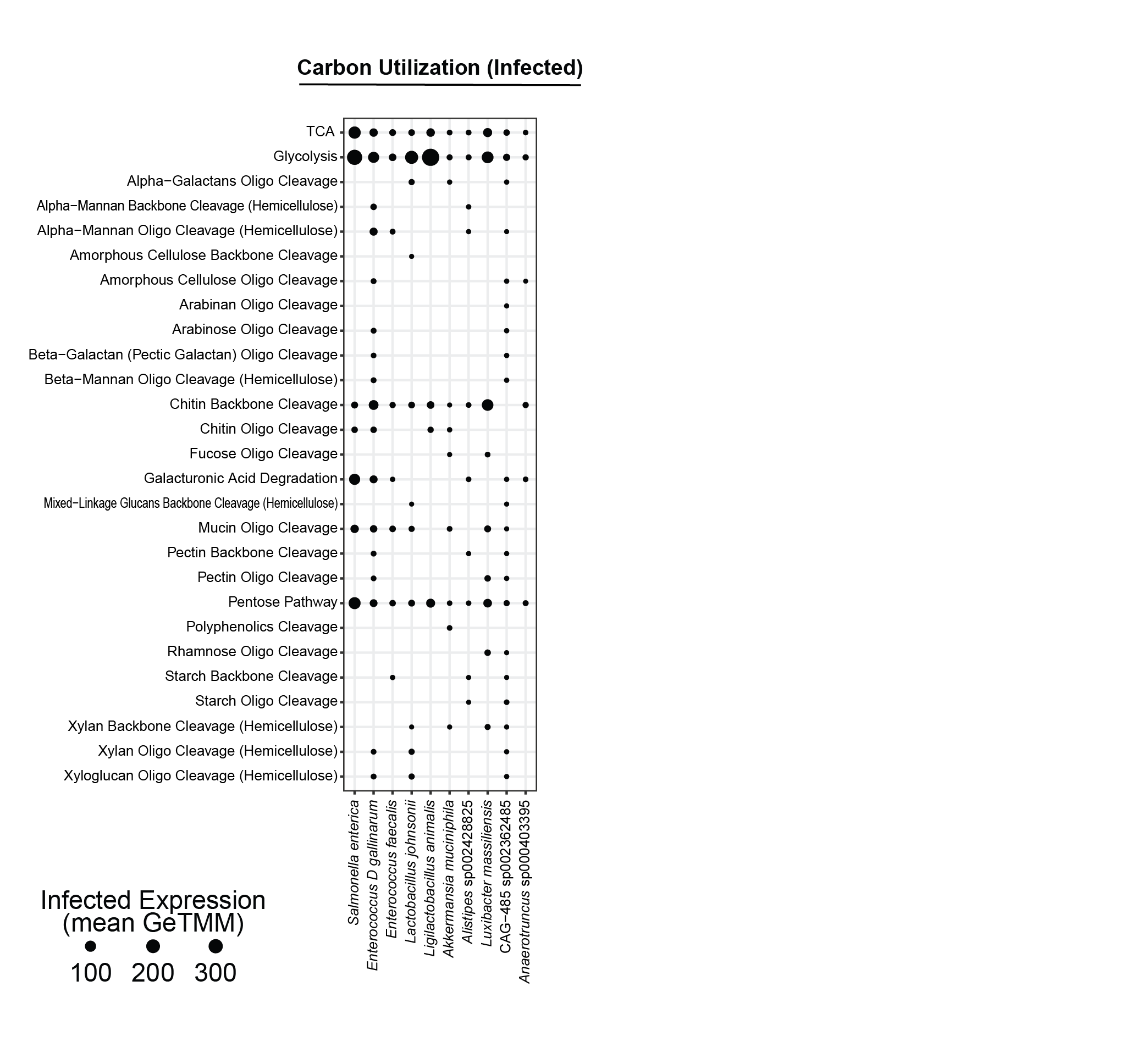
**

**Fig S5: Differential gene expression of genes for energy production show altered metabolic strategies between treatments in prominent infected community members.** Heatmap of actively expressed genes for energy production, including respiration, with select substrate utilization genes that are turned on during infection and are also significantly differentially expressed between treatments. Cell values are GeTMM scaled totals of all genes linked with a particular gene description (y-axis) averaged across samples within a treatment and expressed by an individual taxon (x-axis). Colored boxes on the right indicated gene class.

**
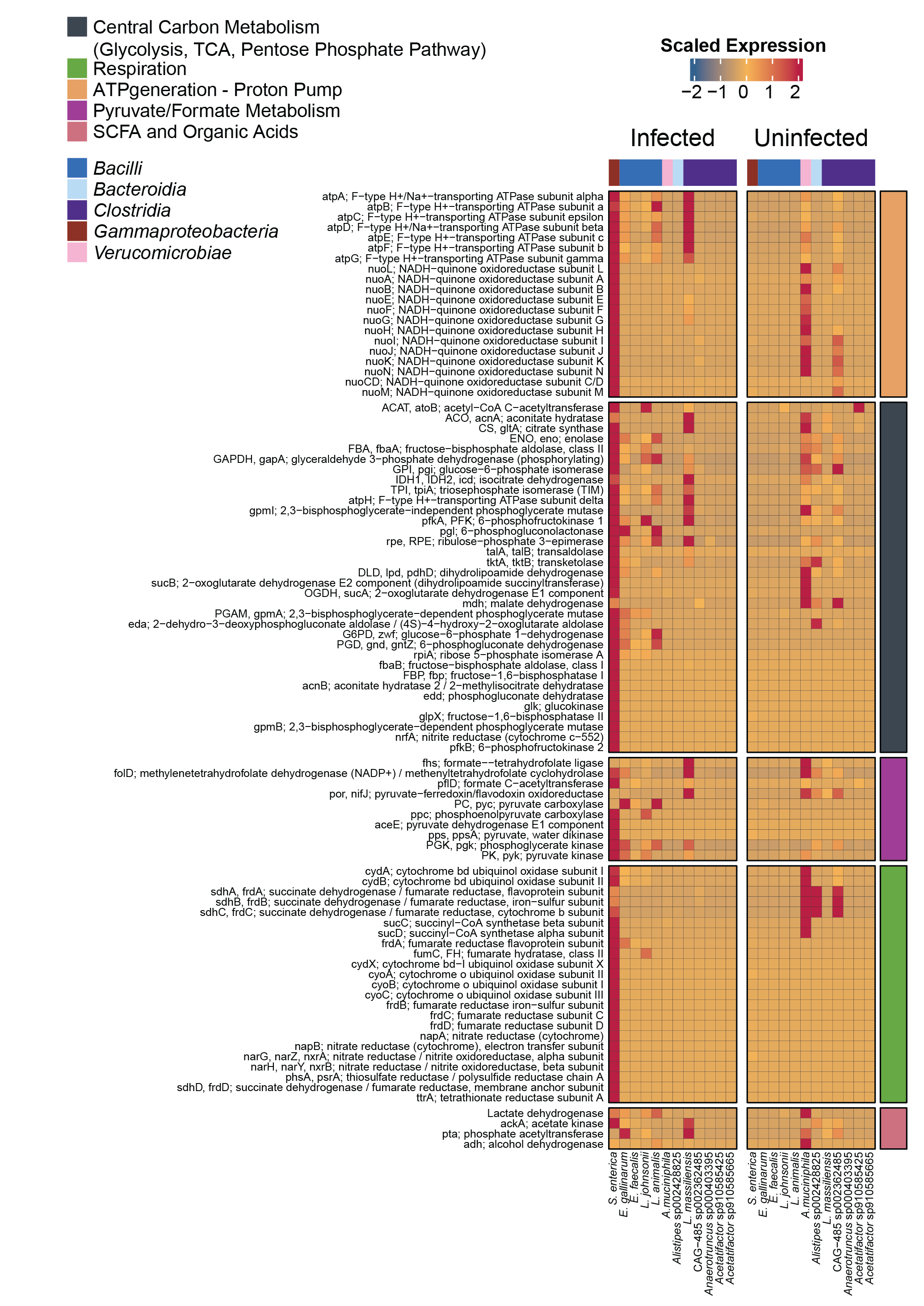
**

**Table S1. Background references for foundational Salmonella physiology from prior murine models, with specific attention to carbon source or electron acceptor use. The table is also provided in excel format in Supplementary Data File Table 1.**

**Legends for Supplemental Data Tables and Files**

**Supplemental Table S1.** Summary of the prior studies that influenced findings in this study in terms of *Salmonella* metabolism.

**Supplemental Data File S1**. Information relating to the 16S rRNA data and general experimental design presented in this manuscript including the following worksheets: (i) inventory of sample data collected for each mouse, (ii) ASV table, (iii) ASV assigned by Silva taxonomy, (iv) lipocalin inflammation data.

**Supplemental Data File S2**. Information related to the metagenome assembled genomes (MAG) used in this study including the following worksheets: (i) MAG mapping via GeTMM normalized counts, (ii) MAG taxonomy assignment, (iii) MAG quality scores, (iv) MAG gene annotations, (v) categorized MAG metabolisms, (vi) curated metabolisms of MAGs, (vii) MAGs linked to 16S rRNA gene content.

**Supplemental Data File S3.** Information related to metatranscriptome data recruited to MAGs in this study including the following worksheets: (i)unfiltered transcripts mapped to CBAJ-DB MAGs with GeTMM normalized counts, (ii) post-filtering mapping (see methods), (iii) Differential expression-Limma differential gene expression performed on metaT counts table, (iv) raw counts table of metaT rarified to 2.5 Gbp of transcript sequencing.

**Supplemental Data File S4**. Information related to untargeted metabolome data reported in this study including the following worksheets: (i) compound annotations and significance between treatments, (ii) compound abundances in each sample.
